# Development of CDK4/6 Inhibitors in Gastrointestinal Cancers: Biomarkers to Move Forward

**DOI:** 10.3390/cimb47060454

**Published:** 2025-06-12

**Authors:** Ioannis A. Voutsadakis

**Affiliations:** 1 Holden Comprehensive Cancer Center, University of Iowa Hospitals and Clinics, Iowa City, IA 52242, USA; ivoutsadakis@yahoo.com or ivoutsadakis@nosm.ca; 2 University of Iowa Carver College of Medicine, Iowa City, IA 52242, USA; 3 Section of Internal Medicine, Division of Clinical Sciences, Northern Ontario School of Medicine, Sudbury, ON P3E 2C6, Canada

**Keywords:** CDK4, CDK6, RB, E2F1, abemaciclib, palbociclib, ribociclib, combination therapy, precision oncology

## Abstract

Targeting the cell cycle has become a focus of cancer research bearing impressive results with the introduction of CDK4/6 inhibitors in the treatment of ER-positive/HER2-negative breast cancers. However, no definitive benefit in other cancers has been observed. In gastrointestinal cancers, despite preclinical studies pinpointing positive effects on cancer inhibition in pre-clinical models, no positive clinical trials have been published with CDK4/6 inhibitors. Several biomarkers have been proposed in breast cancers, where the field is more advanced, and include up-regulations of the inhibited kinases CDK4 and CDK6 and their partner cyclin D as well as the main target of phosphorylation, RB. Up-regulation of Cyclin E, an E2F1/RB regulated gene, also arises as a marker of CDK4/6 inhibition resistance. Signaling from receptor tyrosine kinase pathways through KRAS/BRAF/MEK and PI3K/AKT/mTOR are also implicated in feedback CDK4/6 activation and inhibitors resistance. In gastrointestinal cancers, some of these biomarkers have also proven valuable in predicting sensitivity to CDK4/6 inhibitors and would lead markers to guide clinical development. Modulation of the tumor microenvironment, where immune cells are prominent components, arises as a feature of CDK4/6 inhibition and could be harnessed in therapeutic combinations.

## 1. Introduction

Gastrointestinal cancers represent a significant percentage of the total cancer burden across populations globally [1]. Therapeutic progress in gastrointestinal cancers stems from a better understanding of the genomic alterations that underpin their pathogenesis. This has led to the successful introduction of targeted therapies [2,3,4]. Nevertheless, most patients with metastatic gastrointestinal cancers are still treated with cytotoxic chemotherapies [5,6].

**Figure 1:**
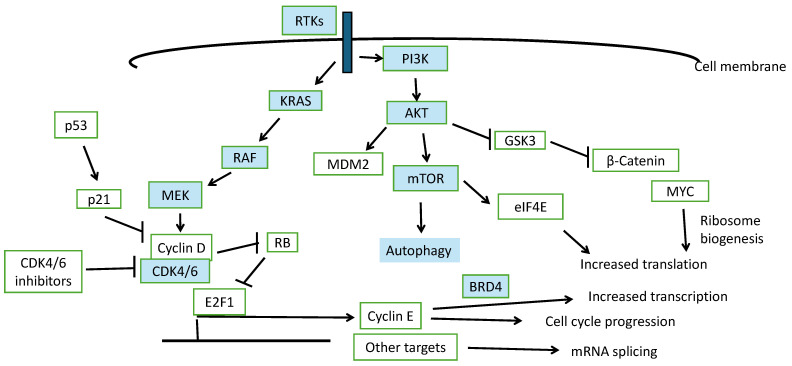
Schematic representation of pathways regulated by CDK4/6 and involved in the cell cycle and potential CDK4/6 inhibitor resistance. Light blue components denote clinically available pharmacologic inhibitors.

The deregulation of the cell cycle is among the hallmarks of cancer [7]. Cyclin-dependent kinases 4 and 6 (CDK4 and CDK6) are serine threonine kinases of the CDK family which encompasses members with roles in cell cycle, transcription and other cell functions [8]. These kinases associate with regulatory proteins called cyclins to phosphorylate substrates mediating their functions [9]. Cyclins of the cyclin D family are the partners that are complex with either CDK4 or CDK6 to promote cell cycle progression at the G1/S junction through phosphorylation and inhibition of protein RB (Figure 1) [10]. Phosphorylated RB dissociates from transcription factor E2F1 which is freed to transcribe genes required for cell cycle progression from G1 phase into initiation of DNA transcription in the S phase. The CDK4 and CDK6 kinase function requires association with Cyclin D and the complex is regulated by mitogenic signals from receptor tyrosine kinase cascades. Both the RAS/BRAF/MEK and the PI3K/AKT signaling pathways activate CDK4/6 [10]. Other signaling cascades activating these kinases include the WNT/β-catenin pathway and, an important player specifically in breast cancer, Estrogen Receptor (ER) signaling [11]. Through the induction of CDK inhibitor p21, tumor suppressor p53 contributes to the regulation of the CDK4/6/Cyclin D complex and the cell cycle [12,13].

Targeting the cell cycle pharmacologically has become a cardinal direction of cancer therapeutic development efforts. These have culminated in the development of specific CDK4/6 inhibitors that have entered clinical practice. Three oral drugs, abemaciclib, palbociclib and ribociclib are currently approved and used, mostly in combination with hormonal therapies, in breast cancer patients, but they have not obtained any approval in other cancer indications [14]. Although the three drugs inhibit the same target, they differ in their pharmacology, efficacy results, approved indications for use and adverse effect profile [15,16].

This review synthesizes biomarker data that could enable rational clinical development of CDK4/6 inhibitors in GI malignancies.

## 2. Methods

A search of the literature was undertaken in the pubmed database (www.pubmed.gov). The focus was on clinical trials and preclinical studies of CDK4/6 inhibitors, either alone or in combination with other drugs, in gastrointestinal cancers. Original articles or reviews on these subjects or studies providing mechanistic insights on the potential benefit of CDK4/6 inhibitors were considered. Search terms were cyclin-dependent kinase 4/6 inhibitors or CDK 4/6 inhibitors or abemaciclib or palbociclib or ribociclib and gastrointestinal cancers or esophageal cancer or gastroesophageal cancer or gastric cancer or pancreatic cancer or colorectal cancer or colon cancer or rectal cancer or biliary cancer or cholangiocarcinoma or hepatocellular carcinoma. Retrieved citation abstracts were reviewed, and full texts were examined and included in the review, if relevant [17]. References of the retrieved articles were manually scanned and also included if deemed relevant. Last database review was performed on 31 March 2025.

## 3. Esophageal and Gastric Cancers

### 3.1. Clinical Trials

Early clinical trials of CDK4/6 inhibitors have been performed in gastroesophageal cancers and potential associated biomarkers of efficacy have been examined (Table 1). A small phase 2 trial of palbociclib in 21 patients with metastatic gastric adenocarcinoma, gastroesophageal junction adenocarcinoma and esophageal adenocarcinomas and squamous carcinomas observed no responses [18]. The median progression-free survival (PFS) was 1.8 months, and the median overall survival (OS) was 3 months. All patients had intact nuclear RB expression by immunohistochemistry and four patients had CCND1 over-expression. In a phase 1 trial with riboociclib that included nine patients with esophageal cancer, no responses were observed and plans for a phase 2 expansion in the disease were abandoned [19]. Therefore, CDK4/6 inhibitor monotherapy was inactive in upper gastrointestinal carcinomas, despite the expression of the target protein of the kinases inhibited, RB. The innate resistance of these cancers to CDK4/6 inhibitor monotherapy may relate to the over-expression of cyclin E, which could continue driving the cell cycle in the presence of CDK4/6 inhibition (Table 2) [20]. Amplification of the cyclin E locus at chromosome 7q21 was observed in about a third of esophageal adenocarcinomas in a study of 116 patients [21]. Patients with the amplification, as well as with over-expression of cyclin D had worse survival than those without these alterations.

### 3.2. Pre-Clinical Data

Knockdown of CDK6 or CDK4 with small interfering RNA or pharmacologic inhibition with palbociclib led to proliferation suppression and inhibition of anchorage-independent survival in esophageal adenocarcinoma cells [21]. Treatment with abemaciclib of rats bearing esophageal adenocarcinoma xenografts, established from three different cell lines, resulted in tumor volume reduction and down-regulation of cyclin D, CDK4, CDK6 and E2F1, compared with rats treated with placebo [22]. In another preclinical study, the methylation status of cyclin inhibitor p16, which is associated with aggressive cancers, was predictive of abemaciclib sensitivity [23]. Among gastric cancer sub-types, MSI-high and EBV-associated cancers displayed the frequent methylation of *CDKN2A*, encoding for p16, and were abemaciclib-sensitive. The groups of genomically stable and chromosome unstable (CIN) gastric cancers had less frequent p16 methyalation, but those with p16 methylation were abemaciclib-sensitive [23]. p16 methylation was also associated with palbociclib sensitivity in an vitro survey of cell lines from various cancers and increasing p16 methylation in lung and gastric cancer cell lines by transfection with a DNA methyltransferase increased their palbociclib sensitivity [24]. Resistance of gastric cancer cells to abemaciclib developing from prolonged exposure to the drug was associated with up-regulation of eukaryotic translation initiation factor eIF4E [25]. Treatment with the antiviral drug ribavirin, an inhibitor of eIF4E, or knockdown of eIF4E was effective in inhibiting resistant cell lines. Ribavirin was effective also in abemaciclib-sensitive cell lines and had a synergistic effect with abemaciclib in abemaciclib-sensitive xenografts in vivo [25]. Synergistic effects were also observed between abemaciclib and the BRD4 inhibitor JQ1 in gastric cancer cells, suggesting a role of inhibiting DNA transcription, in addition to inhibiting translation, to augment the cell cycle inhibiting effect of abemaciclib [26].

Inputs from several pathways culminate in the regulation of the cell cycle [9]. Inhibition of CDK4/6 by palbociclib was associated with up-regulation of cyclin inhibitors p16 and p21, as well as apoptosis inducers Bax and p53 [27]. In addition, Notch was suppressed in cancer cell lines treated with palbociclib in vitro and exposure to the ligand of the Notch pathway Jagged reversed the palbociclib effects, restoring proliferation of these cell lines [27]. The presence of a functional p53 is required for successful treatment of gastric cancer cells with palbociclib to avoid autophagy and senescence, as an adaptive mechanism of resistance [28]. This may relate to the direct interaction of p53 with E2F1 in association with MDM2, which leads to interference with its transcription function [29,30]. Moreover, the interaction promotes the nuclear retention of p53 and triggers the transcription of apoptosis-promoting proteins, acting as a feedback break to unregulated cell cycle [31]. Induction of autophagy was shown in KRAS mutant gastric cancer cell lines exposed to palbociclib and the MEK inhibitor trametinib [32]. The addition of the autophagy inhibitor chloroquine to the palbociclib and trametinib combination enhanced their anti-proliferative effect. Synergy of palbociclib and the multi-kinase inhibitor afatinb was also shown in squamous esophageal carcinoma cells, suggesting that inhibition of feedback activity from upstream kinases is important to enhance the therapeutic effect of CDK4/6 inhibition [33].

The hippo/YAP pathway is a transcriptional inducer of cyclin D, which contributes to the growth effects observed in cells with increased YAP/TEAD transcriptional activity [34]. Activation of YAP/TEAD transcription through transcriptional down-regulation of the hippo regulator LATS2 by transcription factor PAX6 led to CDK4/6 inhibitor resistance in gastric cancer cells [35]. Deregulation of hippo is frequent in gastric cancers associated with intestinal metaplasia and increased transcription activity of YAP/TAZ/TEAD may lead, in addition of promoting intestinal metaplasia, to cyclin D inhibition resistance [36]. Cyclin D is also a target of TAZ in breast cancer stem cells and hippo/YAP/TAZ is a pathway promoting CDK4/6 resistance in breast cancer too, implying a global role of hippo/YAP/TAZ in cell cycle transition regulation [37,38].

**Table 1:** Clinical trials of CDK4/6 inhibitors in gastrointestinal cancers. OR: Objective responses; PFS: progression-free survival; OS: overall survival; DCR: disease control rate; mos: months; wt: wild type; CEA: carcinoembryonic antigen.

Reference	Cancer Type	Phase	Number of Patients	Treatment	Results
Karasic et al. [18]	Gastric/gastroesophageal	2	21	Palbociclib	No OR, PFS: 1.8 mos, OS: 3 mos
Doi et al. [19]	Esophageal	1	9	Ribociclib	No OR
Infante et al. [39]	Colorectal	1	19	Ribociclib	No OR
Sorah et al. [40]	Colorectal KRAS wt	1	10	Palbociclib and cetuximab	No OR, PFS: 1.8 mos, OS: 6.6 mos
Weitz et al. [41]	Mucinous peritoneal GNAS mutant	Personalized cohort	16	Palbociclib	Prolonged stability and CEA responses observed
Chiorean et al. [42]	Pancreatic	Early phase		Abemaciclib with or without LY3023414 versus chemotherapy	Chemotherapy arm (capecitabine or gemcitabine) numerically superior in PFS and DCR
Tang et al. [43]	Pancreatic	Case series	5	CDK inhibitor and trametinib	No OR
Al Baghdadi et al. [44]	Pancreatic and biliary with CDKN2A loss or mutation	Basket trial		Palbociclib	No OR, PFS: 2 mos, OS: 3 mos

**Table 2:** Molecular alterations associated with CDK4/6 inhibitor resistance in gastroesophageal cancers.

Reference	Alteration	Implications
Min et al. [20]	Amplification of Cyclin E	Worse survival when cyclin E amplification or cyclin D over-expression present. Knock-down of CDK4 and CDK6 reverses resistance
Ismael et al. [21]	Over-expression of Cyclin D	
Bae et al. [23]	Methylation of CDKN2A	Methyltransferase transfection increased palbociclib sensitivity
Zha et al. [25]	Up-regulation of eIF4E	eIF4E inhibitor ribavirin or eIF4E knock-down sensitized cells to abemaciclib
Bi et al. [27]	Notch signaling	Jagged antagonized CDK4/6 inhibition
Inoue et al. [29]	p53 dysfunction	P53 function required to prevent autophagy and senescence as mechanisms to CDK4/6 inhibitor resistance
Zhang et al. [35]	YAP/TEAD signaling	YAP activity associated with both gastric intestinal metaplasia and CDK4/6 inhibitor resistance

## 4. Colorectal Cancer

### 4.1. Clinical Trials

CDK4/6 inhibitors have also been studied in early trials that included colorectal cancer patients. No responses were observed with ribociclib monotherapy in the 19 patients with metastatic colorectal cancer of a phase 1 trial that was conducted in patients with various cancer diagnoses [39] (Table 1). A phase 1 trial that enrolled only 10 patients with *KRAS* wild-type metastatic colorectal cancer, who had previously obtained at least 4 months of disease control with cetuximab-based combinations, examined the combination of cetuximab with palbociclib [40]. The disease control rate at 4 months was 20%, with no objective responses observed. The median PFS was 1.8 months, and the median OS was 6.6 months, which did not fulfill the pre-specified criteria for the continuation of the trial [40]. Peritoneal mucinous carcinomatosis with *GNAS* mutations is a neoplasm that has shown sensitivity to palbociclib treatment in a single-arm clinical trial of 16 patients, mostly with an appendiceal primary (2 patients had an unknown primary site and one patient had a pancreatic primary) [41]. Among the 16 patients, 50% had stable diseases at 12 months and the median survival was not reached after a follow-up of 17.6 months. These positive results require confirmation in larger prospective studies. GNAS mutations are observed in about a third of appendiceal cancers and define a group with chemotherapy resistance [45].

### 4.2. Pre-Clinical Data

In a preclinical study in colorectal cancer cells with or without *KRAS* and *PIK3CA* mutations, the combination of ribociclib with alpelisib was more effective than each drug alone in vitro and in vivo in mice xenografts [46] (Table 3). Moreover, the combination led to a simultaneous decrease in the phosphorylation of RB and of targets downstream of PI3K, AKT and S6. Palbociclib with the dual PI3K/mTOR inhibitor gedatolisib was also synergistic in colorectal cancer cell lines and led to decreased S6 phosphorylation [47]. In cells from a colorectal cancer patient with *BRAF* and concomitant *PTEN* mutation that were resistant to cetuximab with encorafenib, the addition of ribociclib led to improved suppression of viability in vitro [48]. The effect of a combination of palbociclib with the MEK inhibitor binimetinib was examined in a study of patient-derived xenografts and was shown to be most effective in xenografts with wild-type *TP53* [49]. In this model, prolonged exposure to the combination was associated with resistance which was reversed by the addition of the SHP inhibitor Navire 13909. The requirement of wild-type *TP53* for the efficacy of CDK4/6 inhibitors may also relate to their efficacy in breast cancers, where ER-positive breast cancers bear mutations of *TP53* only rarely, while triple-negative cancers, which are resistant, have such mutations in 90% of cases [50,51]. Besides colorectal cancer, the requirement of a functional p53 for sensitivity to CDK4/6 inhibition was observed in gastric cancer cells, as discussed above. Other in vitro studies with colorectal cancer cells have shown that the effectiveness of combinations of ribociclib with conventional chemotherapy varies according to the specific chemotherapeutics used and the mutational landscape of the cells [52,53]. The combination of ribociclib with 5FU was synergistic in both *PIK3CA* mutant HT-29 cells, which bear a non-classical *PIK3CA* mutation at codon 449, and in *KRAS* mutant SW480 cells, while ribociclib with irinotecan was synergistic in HT-29 cells but antagonistic in SW480 cells. *KRAS* mutant colorectal cancer cells resistance to palbociclib was associated with increased expression and phosphorylation of cyclin inhibitor p27, mediated by kinase Src [54]. Src kinase inhibitors sensitized cells to palbociclib in this model.

The importance of oncogene MYC in the cell cycle and sensitivity to palbociclib was observed in a study based on transcriptomic and proteomic analysis of organoids established from human colorectal tumors [55]. Organoids that were sensitive to palbociclib showed enrichment in MYC targets and altered fatty acid and lipid metabolism that the authors hypothesized that they were a consequence of MYC activation. MYC activity is frequently increased in colorectal cancers and therefore may become a useful biomarker of CDK4/6 inhibition prediction if it could be clinically measured.

A putative role of the CDK4/6 signaling and the cyclin inhibitor p21 in the immune status of colorectal cancer and the anti-tumor fitness of infiltrating CD4+ T cells was proposed in a study of colorectal cancer in mice [56]. In a model of Rag^-/-^ mice that were immune reconstituted with p21 deleted CD4+ T cells, mice developed more voluminous tumors than mice reconstituted with control CD4+ T cells. The anti-tumor activity of the p21 deleted T cells was improved when they were pre-treated with palbociclib, suggesting that the excess CDK4/6 activity was responsible for the cytotoxic defect. In another study that examined the effect of immune stimulation of macrophages in a colorectal cancer model, blockade of adenosine receptor CD73 with the inhibitor AB680 led to decreased PD-L1 induction and synergized with palbociclib in reducing the growth of colorectal tumors growing in mice [57]. In MMR-deficient gut adenocarcinoma produced by knock-out of MLH1 or MSH2 in mice, abemaciclib was as effective as a murine anti-PD-L1 monoclonal antibody in controlling tumor growth [58]. Abemaciclib exposure led to decreased PI3K/AKT activity in tumor cells and decreased tumor infiltration by regulatory T cells.

Alternative targets of phosphorylation by CDK4/6, besides RB, may also be of potential therapeutic interest. Deubiquitinating enzyme 3 (DUB3) was suggested as a target of CDK4/6 phosphorylation at Serine 41, leading to DUB3 activation [59]. DUB3 deubiquitinates and stabilizes oncogenic transcription factor YAP1, by preventing its proteasome degradation. Inhibition of CDK4/6 with abemaciclib led to increased proteasome-dependent degradation of YAP1, an effect that was also mimicked by mutation of DUB3 at the phosphorylation target codon (S41A) [59]. Therefore, as in gastroesophageal cancers, YAP/TAZ inhibition may be a critical mediator of CDK4/6 inhibitor effects.

**Table 3:** Molecular alterations associated with CDK4/6 inhibitor resistance in colorectal cancers.

Reference	Alteration	Implications
Aslam et al. [46], Lee et al. [47]	Decrease phosphorylation of RB, AKT and S6 following treatment	Combination therapies of CDK4/6 inhibitors and PI3K/AKT pathway inhibitors synergistic
Sorokin et al. [49]	p53 mutations	Combination of palbociclib and binimetinib more effective in p53 wild-type cell
Rambioni et al. [54]	p27 over-expression and phosphorylation	Palbociclib resistance reversed with addition of Src inhibitors
Papacio et al. [55]	MYC activity	Cells with increased expression of MYC targets were more sensitive to palbociclib
Thoma et al. [56]	Increased CDK4 activity in CD4+ T cells	Increased CDK4 activity due to p21 deletion impairs anti-tumor CD4+ T cells function
Wen et al. [59]	YAP stabilization	CDK4/6 inhibition decreases activity of de-ubiquitinase DUB3 and leads to increased proteasome degradation of YAP1

## 5. Pancreatic and Biliary Cancers

### 5.1. Clinical Trials

Abemaciclib as monotherapy or in combination with the dual PI3K/mTOR inhibitor samotolisib (LY3023414) was compared with standard-of-care capecitabine or gemcitabine in an early-phase multicenter clinical trial in patients with metastatic pancreatic adenocarcinoma who had received 1 or 2 previous lines of therapy [42] (Table 1). The two abemaciclib arms were numerically inferior to the standard of treatment arm in DCR and median PFS and no further development was planned. No responses were also observed in five pancreatic cancer patients treated in later line therapy (2 to 6 previous lines of therapy) with the MEK inhibitor trametinib and abemaciclib or palbociclib [43]. In a case study of a pancreatic cancer patient who initially responded but subsequently progressed on trametinib and the autophagy inhibitor hydroxychloroquine, developing c-MYC amplification and CDK4/6 up-regulation, a biochemical response was obtained with the combination of palbociclib with hydroxychloroquine [60]. The importance of lysosomes in CDK4/6 inhibitors resistance was also reported in breast cancer, where destabilizing lysosomes with chloroquine, seremesine, or azithromycin had a sensitizing effect in resistant triple-negative breast cancer cells [61]. Lysosomal trapping of palbociclib becoming more pronounced with increased lysosome biomass was a resistance mechanism, reversed by chloroquine [62]. Palboociclib monotherapy was used in an arm of the Targeted Agent and Profiling Utilization Registry (TAPUR) basket trial which included both pancreatic and biliary cancer patients with *CDKN2A* loss or mutation [44]. There were no responses in either pancreatic or biliary cancer patients included in the trial. Median PFS was less than 2 months and median OS was about 3 months. These results suggest that the requirement for intact CDK4/6/Cyclin D/RB machinery for pharmacologic CDK4/6 inhibitor sensitivity includes intact cell cycle-dependent kinase inhibitors. However, responses have been observed in case reports of patients with loss of CDKN2A/2B when treatment with CDK4/6 inhibitors was combined with multikinase inhibitors [63,64]. In a case study of a metastatic cholangiocarcinoma patient with FGFR2, CDH1 and CDKN2A/2B mutations, after progression on the multi-kinase inhibitor pazopanib, the addition of crizotinib and palbociclib led to a long-lasting response [65]. The tumor in this patient was microsatellite stable (MSS), had a low tumor mutation burden (TMB) of two mutations/Mb and a low expression of PD-L1. In this study, pazopanib was used for its broad kinase-blocking activity, as specific FGFR inhibitors were not available in the patient’s jurisdiction, crizotinib was used based on data for synthetic lethality between ROS kinase inhibition and E cadherin loss and palbociclib was used to inhibit the dysfunction of cell cycle checkpoint produced by the p16 gene mutation.

### 5.2. Preclinical Data

The cell cycle and G1-S phase transition are of a therapeutic interest in pancreatic cancer where the cycle regulator p15, encoded by *CDKN2B* gene is frequently deleted or mutated [66]. In addition, a high frequency of *KRAS*-activating mutations in the disease may activate the cell cycle through the MEK/ERK cascade and mutations in *TP53*, as well as deletions in the *CDKN2A* gene, which has two overlapping reading frames encoding for the p53 regulator p14 and the cell cycle inhibitor p16, may further debilitate regulation of the cell cycle [67]. These alterations are present individually or in combination in most pancreatic adenocarcinomas and create an opportunity for therapeutic manipulation. Along these lines, a study sought to combine palbociclib with knock down of miR-21 a micro-RNA that promotes the cell cycle [68]. The combination was synergistic in blocking proliferation of pancreatic cancer cells in S phase and inducing apoptosis. In another study, the proliferation of pancreatic xenografts was significantly curtailed by the combination of ribociclib and MEK inhibitor binimetinib, and the survival of mice bearing the xenografts was prolonged [69] (Table 4). A similar strategy combining palbociclib with the ERK inhibitor ulixertinib has advanced to an early phase clinical trial [69]. The cell cycle, together with PI3K/AKT signaling, the DNA damage response and repair, and autophagy were confirmed as most important nodes synergizing with CDK4/6 inhibitors in a CRISPR-Cas9 screen of pancreatic cancer cells [70]. A combination strategy employing the HDAC inhibitor panobinostat and abemaciclib showed synergism in vitro in pancreatic cell lines Mia PaCa-2, BxPC-3 and AsPC-1 [71]. The combination decreased proliferation, colony formation and invasion of pancreatic cancer cells more than each drug alone. Another combination strategy that appeared to be synergistic with abemaciclib in pre-clinical pancreatic cancer models was the inhibition of the mRNA stabilizing protein HuR, that stabilizes cyclin D mRNA, and the inhibition of hippo pathway transcription factor YAP1 [72].

Moreover, the combination of abemaciclib with standard gemcitabine and cisplatin chemotherapy was effective in inhibiting biliary cancer cells in vitro and in vivo [73]. The mechanism involved inhibition of autophagy which was a pathway of resistance to abemaciclib monotherapy. The WNT pathway is activated by transcription factor Proline-rich homeodomain/hematopoietically expressed homeobox (PRH/HHEX) and, in co-operation with Notch 3, promoted cell proliferation in cholangiocarcinoma cells and xenografts [74]. Moreover, cells with PRH/HHEX over-expression were sensitive to CDK4/6 inhibition. Sensitivity to palbociclib was also observed in an in vitro and in vivo model of cholangicarcinoma with cyclin D/CDK4/6 pathway activation induced by factor HMGA1 [75]. In this model, palbociclib synergized with a PI3K/mTOR inhibitor in obtaining more sustained inhibition. Synergy was present in vivo in a mouse intrahepatic cholangiocarcinoma model between palbociclib and the mTOR inhibitor MLN0128 (sapanisertib) [76]. In this study, tumors were induced by the injection of Akt and a mutated form of Yap, and synergism was associated with the prevention of the feedback up-regulation of cyclin D, observed with CDK4/6 inhibition. Palbociclib may also decrease the activity of the Akt/mTOR pathway up-stream of Akt by inhibiting IRS2 and TSC2 [77]. Kinase FAK phosphorylates Yap at tyrosine 357, resulting in its activation, and ablation or inhibition of FAK synergized with pallbociclib in inhibiting mouse intrahepatic cholangiocarcinoma [78]. Cyclin D, CDK4 and CDK6 mRNA expressions were up-regulated in cholangiocarcinoma tissues compared with adjacent normal epithelium [79]. However, despite decreasing RB phosphorylation and inhibiting the cell cycle, palbociclib exposure had no effect in apoptosis induction in biliary carcinoma cells HuCCT-1, KKU-213A and KKU-213B. Moreover, palbociclib increased phosporylation of the NF-κB sub-unit p65 and induced the anti-apoptotic proteins cIAP1 and cIAP2 [79]. The combination of palbociclib with the apoptosis inducer SMAC mimetic drug LCL161 was synergistic in cholangiocarcinoma cells growing both in 2D and 3D cultures (Table 4). Synergism was also evident between palbociclib and hinokitiol, a drug that inhibits kinases ERK and p38 in the KRAS pathway [80].

**Table 4:** Molecular alterations associated with CDK4/6 inhibitor resistance in pancreatic and biliary cancers.

Reference	Alteration	Implications
Pancreatic		
Willobee et al. [69], Goodwin et al. [70]	PI3K/AKT signaling, autophagy, DNA damage response	Blocking feedback activation of the KRAS/MEK/ERK pathway with binimetinib or ulixertinib synergized with CDK4/6 inhibitors
Dhir et al. [72]	YAP1	Inhibition of YAP1 activity synergized with CDK4/6 inhibition
Cholangiocarcinoma		
Menapree et al. [79]	cIAP1 and cIAP2 induction	Palbociclib synergized with the SMAC mimetic drug LCL161
Bai et al. [80]	ERK and p38 kinases activity	Palbociclib synergized with ERK and p38 inhibitor hinokitiol

## 6. Hepatocellular Carcinoma

### 6.1. Clinical Data

A case series described patients with hepatocellular carcinomas or mixed hepatocellular–cholangiocarcinomas who progressed on first-line atezolizumab/bevacizumab and were analyzed for targetable lesions [81]. A patient with a mixed hepatocellular- cholangiocarcinoma and amplification of CDK4 was treated with palbociclib and obtained a long-lasting partial response for 16 months [81]. Although a single case can only be the basis for further investigation, together with the preclinical studies below, the data suggest that sub-sets of hepatocellular carcinomas with specific molecular aberrations could be candidates for treatment with CDK4/6 inhibitors, in combination with other relevant targeted therapies.

### 6.2. Pre-Clinical Data

In vitro experiments in hepatocellular carcinoma cell lines disclosed a higher sensitivity to ribociclib in cell lines with high protein expression of RB and low expression of cyclin inhibitor p16, compared with cells that had lower RB expression and high p16 expression [82] (Table 5). Sensitivity to ribociclib was independent of whether the hepatocellular carcinoma cell lines were sensitive or resistant to the multikinase inhibitor sorafenib. In another study, abemaciclib promoted cell cycle arrest in RB-positive hepatocellular carcinoma cell lines, in combination with the kinase inhibitor lenvatinib [83]. Oncogene c-myc was down-regulated with the combination treatment and decreased MAPK and mTOR activity was also observed. Palbociclib resistance was also associated with loss of RB in human hepatocellular carcinoma cell lines [84]. In a mouse model of hepatocellular carcinoma with p53 mutations and RB loss, forced expression of RB sensitized tumor cells to palbociclib and a similar phenotype was observed when a non-phosphorylatable form of RB was expressed in the tumors [85]. Either palbociclib treatment or the presence of the non-phosphorylatable form of RB led to activation of NF-κB due to phosphorylation of inhibitor I-κB kinase IKKα/β. An inhibitor of this kinase abrogated NF-κB activation and synergized with palbociclib in inhibiting tumors [85]. Kinase AMPK was suggested as a target of inhibition for palbociclib, independently of CDK4/6, in hepatocellular carcinoma cells, while the two other inhibitors, abemaciclib and ribociclib had no inhibitory effect in this kinase [86]. Another target of palbociclib, which is CDK4/6 dependent, is the mutant form of p53 at position R249S which creates a new phosphorylatable site [86]. Phosphorylation of the newly substituted serine at this position by CDK4/6 creates a binding site for MYC and increases ribosomal biogenesis in hepatocellular carcinoma cells. Palbociclib in combination with a drug that restores the conformation of mutant p53 reversed these effects [87]. p53 is mutated in about a third of hepatocellular carcinomas and the specific R249S mutation constitutes about 5% to 10% of the mutations in the tumor suppressor [88].

## 7. Discussion

The dearth of data available from different cancer settings and experimental models pinpoints the critical regulation of the cell cycle and its deregulation, as a valid therapeutic target. However, both pre-clinical studies and clinical trials imply that the deregulation of CDK4/6/Cyclin D/RB node has variable repercussions dependent on the genomic micro-environment of a given cancer. Therefore, the pharmacologic inhibition of CDK4/6 shows variable, and in many cases no efficacy, depending on feedback loops and parallel running inputting pathways. These promote primary and secondary resistance. Combination treatments appear to be the best promise for advancing the field in gastrointestinal tract cancers and potentially in other cancers. Indeed, the success of CDK4/6 inhibitors in ER-positive/HER2-negative breast cancers has been critically based on the concomitant inhibition of ER, as a crucial inputting pathway in these cancers. In these breast cancers, new generations of ER inhibitors have already entered clinical trials in combinations with CDK4/6 inhibitors to circumvent resistance related to altered ER signaling [89]. The inhibition of CDK4/6 promotes escape mechanisms in the cell cycle control machinery and among these mechanisms, CDK inhibitor p27 expression levels featured prominently in resistance development [90]. Decreased levels of p27 were associated with resistance, which could be circumvented by concomitant CDK2 inhibition.

### 7.1. Alternative E2F1 Targets

In addition to targeting genes involved in the promotion of the cell cycle, E2F1 induces genes promoting apoptosis and DNA damage response and repair, such as TP73, pro-apoptotic members of the BCL2 family, BRCA1, RAD52, MSH2 and MLH1 [91,92]. These additional targets may act as a safety net to curtail the proliferation of cells under genomic stress. TP73 could be a critical factor in apoptosis execution in this network and may be instrumental in cells with a dysfunctional p53, which may explain the requirement of intact p53 for CDK4/6 inhibitor sensitivity [93]. Apoptosis dependent on p53 is also observed following induction of E2F1 [91]. In addition, E2F1 and p53 cooperate in modulating metabolic pathways gene expressions. For example, in pancreatic cancer cells with KRAS G12D mutations, the activity of the axis up-regulates ubiquitin-conjugating enzyme UBE2T and modulates the pentose phosphate pathway, which is important for the carbon metabolism of cancer cells [94].

E2F1 has also functions affecting alternative mRNA splicing, which are modulated by specific arginine methylation, mediated by methyltransferase PRMT5 [95]. This post-translational modification leads to an entire set of regulations influenced by E2F1. Therefore, inhibiting E2F1 function through CDK4/6 inhibitors has repercussions beyond the cell cycle. Arginine methylation of E2F1 modulates also the transcription of peptides from long non-coding RNAs (lncRNAs) that can be presented on cell surfaces associated with MHC I molecules and elicit an immune response [96]. lncRNA-derived peptides produced CD8+ T lymphocyte infiltration to tumors and delayed growth.

### 7.2. Immune Synergy

Besides cardinal ramifications for the cell cycle inhibition, CDK4/6 inhibitors have anti-tumor immunity-promoting effects which are derived from the inhibition of E2F1 [97]. E2F1 inhibition results in the activation of transcription of endogenous retroviral elements in tumor cells and in triggering the interferon pathway as a response to the presence of cytoplasmic double-strand RNA. In addition, E2F1 inhibition in regulatory T cells down-regulates their activity allowing for increased unhindered anti-tumor effect of cytotoxic T cells [97]. Palbociclib treatment increased T cell infiltration in the tumor microenvironment [98]. CDK4/6 inhibitors have salutary effects in developing anti-tumor immunologic T cell memory [99]. Activation of T cells by CDK4/6 inhibitors synergized with immune checkpoint inhibitors in a syngeneic mouse model in vivo [100]. A gene set enrichment analysis in hepatocellular carcinoma has revealed CDK4 expression to be reversibly correlated with cytotoxic lymphocyte infiltration [101]. In addition, CDK4 expression correlated with expression of the inhibitory receptor of phagocytosis CD47 in immunohistochemistry sections from hepatocellular carcinomas [102]. DNA damage induced by palbociclib but also by anti-mitotic agents, such as paclitaxel and Aurora kinase inhibitors led to cytoplasmic DNA leak and activation of STING (Stimulator of interferon genes), resulting in cytokine production and immune cell infiltration [103]. This effect was intensified by hypoxia, which induced transcription factor HIF and up-regulated STING. In a mouse model of hepatocellular tumor-initiating cells, treatment with palbosiclib reduced tumor development and promoted a senescence-associated secretory phenotype with induction of chemokines CXCL10 and CCL2 and attraction of tumor-suppressing macrophages [104]

### 7.3. Non-Canonical CDK4/6 Targets

Other targets of phosphorylation of CDK4 and CDK6 may also be of therapeutic interest in cancer, including transcription factors FOXM1, SMAD3 and NFAT4 [105,106,107,108]. FOXM1 activation after phosphorylation by CDK4/6 results in G1/S phase progression and protection from senescence [105]. CDK4 also phosphorylates the TGFβ pathway transducer SMAD3 and this phosphorylation inhibits the SMAD3 ability to activate cell cycle inhibitor p15 [106]. Another target transcription factor of CDK4/6 is NFAT4, which is repressed by CDK4/6 phosphorylation, resulting in T cell activation suppression [98]. De-repression of NFAT by palbociclib or another CDK4/6 inhibitor, trilaciclib led to T cell activation and synergized with immune checkpoint inhibitors in an organotypic tumor spheroid culture model and in syngeneic tumors in mice. These results further support the potential for CDK4/6 inhibition in combination with other approved drugs in diverse tumors. Biomarkers of response will be critical for further development of CDK4/6 inhibitors in gastrointestinal and other cancers [108].

### 7.4. Limitations of Pre-Clinical Models

Although the pre-clinical models provide directions for translation to the clinic there are limitations in their direct clinical relevance that stem from the differences in each model from the human in vivo tumor environment, which is difficult to fully capture. Models based on cell lines have several limitations, as they do not address the tumor heterogeneity that develops during the natural history of human cancers. Moreover, both in vitro models and in vivo models of human cell line xenografts in immunocompromised mice provide limited insights into the role of the immune system in human cancers. This may be an important handicap of these models for the investigation of the role of CDK4 and CDK6 and their inhibition as an anti-cancer strategy, given that, as discussed, inhibitors of CDK4/6 affect immune processes. A further degree of complexity in the study of CDK4/6 inhibition in pre-clinical models is encountered in experiments that employ combinations of drugs or other interventions. As combinations of CDK4/6 inhibitors with other drugs appear to be necessary for meaningful clinical activity and for the prevention of activation of feedback resistance loops, devising valid combination treatment models would be a major step forward.

### 7.5. Lessons from Previous Clinical Trials and Future Directions

Despite the limitations of the pre-clinical models and the limited success of clinical trials of CDK4/6 inhibitors in gastrointestinal cancers so far, there is a well-founded hope that these drugs, in combination with other rational targeted therapies, could benefit sub-sets of patients. Pathologic and molecular biomarkers to define these sub-sets will be instrumental in the successful development of CDK4/6 combinations in gastrointestinal cancers. Previous early trials of CDK4/6 inhibitors in gastrointestinal trials have not adequately selected for cancers with dependence on the targeted node. This is due to two main reasons. First, the prevailing model of early trials focuses more on safety and efficacy is less important. Second, the potential biomarkers identified from pre-clinical models had not been analytically validated for use in clinical specimens and the best method for detection of alterations to correlate with CDK4/6 inhibitor sensitivity has not been fully elucidated. For example, whether the nuclear expression of RB protein in IHC sections, as was used in the clinical trial of palbociclib in gastric and esophageal cancers, adequately predicts activity of the inhibitor or correlates with genomic deletions is unknown [18]. Other biomarkers, such as cyclin D, cyclin E and p21 suffer from the same uncertainties. In addition, most studies focus on a limited number of biomarkers, whereas a more comprehensive panel may better tackle the pertinent molecular landscape to accurately predict sensitivity and resistance. The TAPUR trial cohort of *CDKN2A* mutation or loss required only alterations of this gene for inclusion and treatment with palbociclib, whereas the status of other genes of the CDK4/6/Cyclin D/RB node was not examined [44]. The status of potential biomarkers beyond the core target node, such as p53 was also not a requirement for inclusion or a stratification factor.

It is conceivable and even probable that only specific sub-types of each primary gastrointestinal cancer would be innately sensitive to CDK4/6 inhibition, while others would be more resistant, as suggested by the example of MSI- and EBV-related gastric cancers versus CIN and genomically stable gastric cancers, discussed in a previous section. The mechanism suggested to be the basis of this differential CK4/6 inhibitor sensitivity of gastric cancer sub-types, p16 methylation, may serve as a biomarker to guide inclusion to a trial of CDK4/6 inhibitor as treatment in gastric cancers or other gastrointestinal trials. In this basket trial concept only patients with p16 hyper-methylated cancers would be included, maximizing the probabilities of demonstrating a positive signal. Another rational combination based on preclinical data could test the combination of a CDK4/6 inhibitor with a PI3K inhibitor in gastrointestinal cancer patients stratified according to the level of cyclin E expression. The observed salutary effects of CDK4/6 inhibition on anti-tumor immunity could be the basis for a triple combination regimen associating a CDK4/6 inhibitor, an immune checkpoint inhibitor and a BRD4 inhibitor in colorectal cancer. In this trial concept, the invigorated immune system could act in synergy with increased antigen presentation by tumor cells stressed due to transcription defects induced by interference with the BRD4 function. Finally, additional trials to follow on successful initial results are required in the case of appendiceal carcinomas with GNAS mutations and to further elucidate the mechanisms of sensitivity or resistance of these cancers to CDK4/6 inhibitors.

## Figures and Tables

**Table 5:** Molecular alterations associated with CDK4/6 inhibitor resistance in hepatocellular carcinoma.

Reference	Alteration	Implications
Reiter et al. [82]	Protein expressions of RB and p16	Higher protein expression of RB and low expression of p16 associated with ribociclib sensitivity
Bollard et al. [84]	RB loss	Palbociclib resistance
Sheng et al. [85]	Mutated non-phosporylatable RB	Palbociclib sensitivity
Sheng et al. [85]	NF-κB activation	Palbociclib resistance reversed by kinase IKKα/β inhibitor
Wang et al. [87]	p53 mutation R249S	Up-regulation of MYC activity is reversed by palbociclib and p53 modulator drug

## Abbreviation

CDK4	Cyclin dependent kinase 4
CDK6	Cyclin dependent kinase 6
E2F1	E2F transcription factor 1
RAS	Rat sarcoma oncogene
RAF	Rapidly accelerated fibrosarcoma oncogene
MEK	Mitogen activated protein kinase kinase
PI3K	Phosphatidyl-inositol 3 kinase
AKT	AKT serine/threonine kinase 1
CCND1	Cyclin D1 gene
eIF4E	Eukaryotic translation initiation factor 4E
BRD4	Bromodomain
Bax	BCL2 associated X protein
YAP	Yes associated protein
TAZ	Transcription co-activator with PDZ binding motif
TEAD	TEA domain family 1
LATS2	Large tumor suppressor kinase 2
PAX6	Paired box family transcription factor 6
MLH1	MutL homolog 1
MSH2	MutS homolog 2
PFS	Progression-free survival
OS	Overall survival
DCR	Disease control rate
